# How do health professionals learn their communication skills? Lessons for SBI implementation

**DOI:** 10.1186/1940-0640-7-S1-A1

**Published:** 2012-10-09

**Authors:** Niamh Fitzgerald

**Affiliations:** 1 Create Consultancy Ltd. and the Robert Gordon University, Glasgow, Scotland, UK

## 

Although the vital components of effective screening and brief intervention (SBI) are unknown, many studies have defined SBI as including various elements of effective communication. If SBI is to become part of routine practice, it is likely that practitioners will need to be comfortable with a high level of communication and a less authoritarian style. This study explored how staff across the National Health Service learn and develop patient communication skills, attitudes, and behaviors. Fifty-two semi-structured, in-depth, qualitative telephone interviews were carried out to saturation point. Participants included junior and senior doctors, nurses and allied health professionals in cancer, heart disease, respiratory, palliative and mental health care. Interviews were recorded, transcribed in full, and analyzed thematically. Development of communication skills mainly relied on individual practitioners reflecting on their own practice. Few external processes or triggers to encourage or support this among staff were reported. Much emphasis was placed by interviewees on learning by observing and modeling others. Again, this was generally informal and not actively supported or developed. Overall, managers or supervisors reported addressing communication skills with staff only if there was a patient complaint or obvious problem. There were few mechanisms for continuous improvement of skills. Formal appraisals of experienced staff tended to focus on inter-professional versus patient communication. In frontline health care, there is little overt emphasis on developing how well practitioners communicate with patients, especially as practitioners become more senior. Aligning SBI initiatives with initiatives to develop generic communication abilities may have mutual benefits, although there are challenges to overcome.

